# Effect of Si on Marine Corrosion Behavior of Austenite Low-Density Steel

**DOI:** 10.3390/ma19061182

**Published:** 2026-03-17

**Authors:** Yuhe Huang, Shuize Wang, Jiahao Qiang, Hui Wang, Jun Lu

**Affiliations:** Institute for Carbon Neutrality, University of Science and Technology Beijing, Beijing 100083, China; huangyuhe@ustb.edu.cn (Y.H.); wangshuize@ustb.edu.cn (S.W.); 18500713877@139.com (J.Q.); wanghui04@ustb.edu.cn (H.W.)

**Keywords:** austenite low-density steel, marine corrosion, surface oxidation, phase composition, element enrichment

## Abstract

To address the high-salinity and hyper-humid thermal environment of tropical oceans and meet industrial demands for high strength and lightweight, austenitic low-density steel was developed as a novel corrosion-resistant steel. A 3.5 wt.% NaCl solution was used to simulate the marine environment to study the effect of Si on the corrosion behavior of this steel. Scanning electron microscopy (SEM), transmission electron microscopy (TEM), X-ray photoelectron spectroscopy (XPS), X-ray diffraction (XRD) and electron probe microanalysis (EPMA) were employed to characterize the microstructures and corrosion behaviors of two test steels, as well as the phase compositions and element distributions of corrosion products after polarization and cyclic immersion accelerated corrosion tests. The results show that a dense oxide film initially forms on the steel surface in 3.5 wt.% NaCl solution at the early corrosion stage. Si addition induces SiO_2_ formation and promotes Al conversion to Al_2_O_3_, enhancing oxide film compactness and inhibiting matrix atom outward diffusion and Cl^−^ inward penetration. With prolonged corrosion, the oxide film is dissolved or broken, forming a dense rust layer dominated by Fe_3_O_4_, Fe_2_O_3_ and FeOOH. Si enriches in the inner rust layer adjacent to the matrix and pitting cavities, inhibiting pitting deepening and promoting γ-FeOOH to α-FeOOH transformation, thus improving the steel’s corrosion resistance.

## 1. Introduction

Marine platforms, as essential infrastructure for offshore oil and gas exploitation, offshore wind power development, and other marine engineering activities, play an irreplaceable role in energy development, resource transportation, environmental monitoring, and the safeguarding of national maritime defense security [1,2]. According to their connection with the seabed, marine platforms can be classified into fixed platforms and floating platforms, which are, respectively, suited to different marine environments and operational requirements [3,4,5,6]. As marine engineering advances toward deeper and more remote offshore environments with increasingly complex service conditions, the performance requirements for steels used in marine platforms continue to rise, demanding higher strength, longer service life, and superior environmental resistance [7,8,9,10]. Harsh marine corrosion environments characterized by high salinity, high humidity, and elevated temperatures create highly corrosive conditions. Under such circumstances, the synergistic effects of multiple corrosion factors can accelerate the corrosion-induced degradation of platform steels, thereby compromising platform safety and service life and increasing costs and safety risks. Consequently, more stringent requirements are imposed on the corrosion resistance of steels used for marine platforms [11,12].

Lightweight design, long service life, and high specific strength are the core developmental requirements for steels used in marine platforms at present [13,14]. Marine platform structures are massive in scale, and the self-weight of the equipment directly affects load-bearing capacity, construction difficulty, and operational costs. Reducing material density and increasing specific strength can decrease structural weight while maintaining high strength and reliability, thereby enabling lightweight platform design. Meanwhile, a long service life can reduce the frequency of maintenance and replacement caused by corrosion-induced material failure, lower operation and maintenance costs, and ensure the long-term stable service of marine equipment. Austenitic low-density steel, as a novel high-performance structural steel, is well aligned with the above development requirements. Its key advantages stem from its relatively low density, high specific strength, and excellent corrosion resistance. These attributes help overcome major limitations of conventional marine steels, including high density, low specific strength, and poor corrosion resistance in harsh marine environments. As a result, it is a promising material for lightweight, long-service-life marine platforms.

Low-density Fe–Mn–Al–C austenitic steels, developed as low-cost alternatives to Fe–Cr–Ni–C stainless steels since the 1980s and 1990s, have attracted considerable attention, primarily owing to the pronounced beneficial effects of Mn and Al on the mechanical properties and oxidation resistance of these materials. Among these elements, Al exhibits a particularly significant weight-reduction effect: for every 1 wt.% increase in Al content, the material density can be reduced by approximately 1.3% [15]. In addition to Al, Si is also an important alloying element for reducing material density. Si provides solid-solution strengthening, thereby enhancing material strength, while simultaneously increasing the activity of carbon in austenite, hindering the nucleation and growth of carbides, suppressing carbide formation, and promoting carbon enrichment in untransformed austenite, which significantly improves the stability of retained austenite [16]. Moreover, Si possesses high oxide stability and can form a dense SiO_2_ oxide film during high-temperature oxidation, effectively reducing the corrosion rate of the material; it can also shift the steady-state potential in the negative direction, facilitating the transition of metallic materials into a stable passive state [17]. In addition, the presence of Si in steel, together with fine grains, can promote the continuity of the Al_2_O_3_ oxide film, whereas a discontinuous alumina layer does not provide effective corrosion protection [18]. Furthermore, during oxidation, Si oxidizes at a faster rate than Al, enabling the rapid formation of an oxide film.

At present, studies on the role of Si in low-density Fe–Mn–Al–C steels mainly focus on its contributions to mechanical properties and high-temperature oxidation behavior, while systematic investigations into the effects of Si on long-term corrosion behavior and the evolution mechanisms of microscopic passive films under simulated harsh marine environments remain lacking. These limitations result in an insufficiently close linkage between alloy composition design and actual severe service conditions, thereby restricting the reliable application of this class of steels in marine platforms. In chloride-rich marine environments (e.g., seawater immersion or marine atmospheres), the corrosion response of Fe–Mn–Al–(Si) low-density steels is governed by the stability and chemistry of the surface film and corrosion products, and is often evaluated in 3.5 wt.% NaCl as a laboratory surrogate [19,20]. Although widely adopted to stabilize austenite in many Fe–Mn–Al–C lightweight alloys, Mn can lower corrosion resistance because Mn tends to form less protective/unstable oxides and promotes more active dissolution in chloride media. By contrast, Al can enhance corrosion protection when it enriches the passive film and contributes to compact Al_2_O_3_-based layers (or Al-enriched oxides), which strengthens passivation and delays chloride-induced breakdown in Fe–Mn–Al–C lightweight alloys [21]. Silicon typically improves resistance to chloride attack mainly as a film/rust modifier: it can refine the passive layer and raise the pitting potential (expanding the passive region), while also promoting SiO_2_-containing network structures and altering ionic selectivity and elemental partitioning within the oxide/rust layer [22]. Recent composition-design work on Fe–Mn–Al–Cr–Si–Mo–C lightweight steels further indicates that coupling Mn-based austenite stabilization with Al/Si-assisted film stabilization can significantly shift polarization behavior and corrosion mechanisms in 3.5 wt.% NaCl, underscoring the need for balanced alloy design for marine service [23].

In this study, austenitic low-density steel was selected as the research object, and a 3.5 wt.% NaCl solution was used to simulate the marine environment. The corrosion resistance of the material was evaluated through electrochemical experiments, in which potentiostatic polarization tests were employed to simulate the initial stage of corrosion under marine conditions, while a periodic immersion accelerated corrosion test was conducted to simulate long-term corrosion behavior in a marine environment. The effects of Si addition on the oxide film compactness, elemental distribution, phase constitution of corrosion products, and surface morphology of derusted steel in a marine environment were systematically investigated. The influence of Si addition on the corrosion behavior of austenitic low-density steel in a marine environment was elucidated, and the corrosion mechanism of Si-containing low-density steel under marine conditions was clarified, providing a theoretical basis and technical guidance for engineering applications in related fields.

## 2. Materials and Methods

### 2.1. Materials

Two austenitic low-density steels were designed in the laboratory, one without Si and the other containing 0.8 wt.% Si; the chemical compositions of the experimental steels measured by chemical method are listed in Table 1. The steel was produced by vacuum smelting and casting (VIM-25, Beijing Research Institute of Mechanical and Electrical Technology Co., Ltd., Beijing, China). Prior to forging, the steels were homogenized at 1150–1200 °C for 12 h to prevent Mn and Al segregation. After homogenization, forging was carried out, and the forged billets were reheated in a furnace to 1150 °C, held for 2 h, descaled, and then subjected to rolling. Following five passes of hot rolling, with a final rolling temperature of no less than 950 °C, the thickness was reduced from 60 mm to 12 mm, after which the plates were water-cooled to room temperature. The density of the two test steels were measured by the Archimedes method as: 0Si steel was 6.83 g/cm^3^, while that of the 0.8Si steel was 6.80 g/cm^3^. For EBSD characterization, the specimens were prepared sequentially by mechanical grinding (using SiC papers from 250# to 2000#), mechanical polishing, and vibratory polishing (VibroMet 2, Buehler, Lake Bluff, IL, USA) with 50 nm SiO_2_ particles. Tensile specimens were prepared in accordance with GB/T 228.1-2021 [24] (specimen diameter: 10 mm; gauge length: 50 mm). Impact specimens were prepared as standard Charpy specimens in accordance with GB/T 229-2020 [25].

### 2.2. Electrochemical Experiments

Electrochemical experiments were conducted to evaluate the intrinsic corrosion resistance of the experimental steels. The specimens for electrochemical testing were prepared solely by mechanical grinding up to 2000# grit. Electrochemical corrosion tests were carried out in a 3.5 wt.% NaCl solution using an Reference 600+ electrochemical workstation (Gamry Instruments, Warminster, PA, USA). A traditional three-electrode system was used for electrochemical measurements, in which a saturated calomel electrode (SCE) was used as reference electrode, a platinum plat was used as counter electrode, and the test steels were used as working electrode, the working electrode area was 0.785 cm^2^. Prior to testing, the specimens were immersed for 30 min to allow the electrode potential to stabilize, which was taken as the open-circuit potential (OCP). Electrochemical impedance spectroscopy (EIS) measurements were performed over a frequency range from 10^5^ Hz to 0.01 Hz, with an AC perturbation amplitude of ±10 mV, under open-circuit potential conditions. Potentiodynamic polarization (PDP) curves were recorded in the potential range of −1.5 to 0.5 V at a scan rate of 1 mV/s. Each sample is tested three times to ensure data reliability. All electrochemical tests were carried out at a constant temperature of 25 ± 1 °C, with the ambient relative humidity controlled at 45–55% RH.

### 2.3. Potentiostatic Polarization Tests

Oxide films were prepared by potentiostatic polarization tests. Prior to the experiments, the specimens were ground to 3000# grit using abrasive papers and thoroughly cleaned with deionized water to remove surface debris. The tests were relative to the OCP, before potentiostatic polarization, the samples were cathodically polarized at −1.0 V_SCE_ for 300 s to remove the air-formed oxide film, followed by polarization at −0.5 V_SCE_ for 7200 s to form an oxide film on the surface. After polarization, the samples were immediately rinsed with deionized water, dried with absolute ethanol, and then vacuum-sealed to prevent surface oxidation. Surface morphology characterization and compositional analysis were performed using a scanning electron microscope (FE-SEM, Mira 3 LMS, TESCAN, Brno, Czech Republic) equipped with energy-dispersive X-ray spectroscopy (EDS, Ultim Max 170, Oxford Instruments, Abingdon, UK). The phase composition of the oxide film surface was analyzed by X-ray photoelectron spectroscopy (XPS, Thermo Scientific ESCALAB 250Xi, Thermo Fisher Scientific, Waltham, MA, USA); argon ion sputtering was employed, with spectra collected after etching every 6 nm in depth, to a total etching depth of 30 nm, in order to examine the variation in elemental distribution with oxide film depth. Transmission specimens were prepared by focused ion beam (FIB, Helios 5 UC, Thermo Fisher Scientific, Waltham, MA, USA) milling, and the cross-sectional morphology and compositional features of the oxide films were observed using a transmission electron microscope equipped with EDS (FE-TEM, Talos F200X, Thermo Fisher Scientific, Waltham, MA, USA).

### 2.4. Periodic Immersion Accelerated Corrosion Test

The experimental steels were machined into two specimen sizes: large specimens with dimensions of 50 mm × 25 mm × 3 mm (length × width × thickness) and small specimens with dimensions of 11 mm × 10 mm × 3 mm (length × width × thickness). A circular hole with a diameter of 2.5 mm was machined at the center of the width side of each specimen for suspension during testing. For each experimental condition and each corrosion cycle, three parallel large specimens were prepared to calculate corrosion weight loss, and the scraped rust powders were used for phase analysis; meanwhile, three parallel small specimens were prepared for morphological observation and elemental distribution analysis. Prior to the periodic immersion test, all specimens were surface-machined and ultrasonically cleaned with ethanol and deionized water to remove surface contaminants. A 3.5 wt.% NaCl solution was selected to simulate the marine environment. To further accelerate the corrosion process, the corrosion medium temperature was set to 45 °C, the drying temperature to 70 °C, and the relative humidity to 70%, in order to simulate a more severe high-humidity and high-temperature marine corrosion environment. Before the periodic immersion corrosion test, all large specimens were weighed using a precision electronic balance to record the initial mass for subsequent weight-loss measurements. After the completion of the corrosion tests, the specimens were removed and the surface rust layers were eliminated using a chemical derusting method; the derusting solution consisted of 500 mL HCl + 3.5 g C_6_H_12_N_4_ (hexamethylenetetramine) + 500 mL H_2_O. The derusting process was conducted at room temperature (25 °C) with ultrasonic agitation to facilitate the removal of surface-adhered corrosion products. The corrosion durations were 72, 144, 216, 288, and 360 h. The macroscopic morphologies of the corroded specimens were recorded using a digital camera (EOS 90D, Canon Inc., Tokyo, Japan). The phase composition and chemical composition of the rust layers were analyzed by X-ray diffraction (XRD, X’Pert PRO MPD, Malvern Panalytical, Almelo, The Netherlands) and X-ray photoelectron spectroscopy (XPS, Thermo Scientific ESCALAB 250Xi, Thermo Fisher Scientific, Waltham, MA, USA), respectively. The cross-sectional morphology of the rust layers and their elemental distributions were characterized using an electron probe microanalyzer (EPMA, JXA-8530F Plus, JEOL Ltd., Tokyo, Japan). The surface morphology of the derusted specimens was observed using a three-dimensional laser confocal microscope (OLS5100, Olympus Corporation, Tokyo, Japan), and the pit depths were statistically analyzed.

## 3. Results

### 3.1. Microstructure and Mechanical Properties

**Figure 1:**
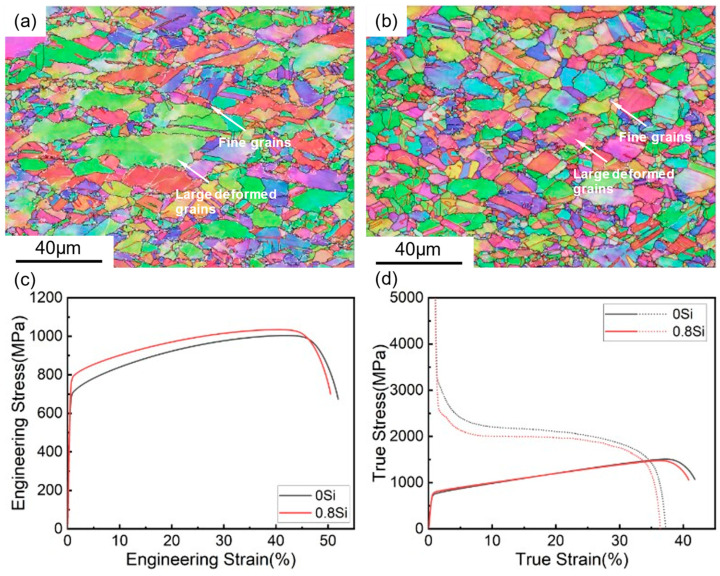
Microstructure and stress curve of experimental steel. (**a**) 0Si; (**b**) 0.8Si; (**c**) stress–strain curves; (**d**) work-hardening curves.

Figure 1 presents the microstructural morphologies and tensile curves of the two experimental steels. The results indicate that the addition of 0.8 wt.% Si has almost no effect on the microstructural morphology of the austenitic low-density steel; both hot-rolled low-density steels exhibit a single-phase austenitic microstructure. The austenite grain size is inhomogeneous, consisting of large deformed grains formed by thermal deformation during the rolling process, as well as fragmented fine grains. As a solid-solution element, Si enhances the material strength through solid-solution strengthening. It suppresses carbide precipitation and promotes carbon enrichment and stabilization of austenite [26]. Consequently, the 0.8Si steel shows a slight increase in ultimate tensile strength, accompanied by a slight decrease in elongation after fracture and total elongation. The mechanical properties of the two experimental steels are summarized in Table 2, including Yield strength (Rp_0.2_), tensile strength (R_m_), elongation after fracture (A_50_), uniform elongation (A_g_), impact energy at 0 °C (KV_2_, 0 °C), specific strength (Rm/ρ). The 0Si steel exhibits an ultimate tensile strength of 1010 MPa, an elongation after fracture of 51.9%, and an impact energy at 0 °C of 148.2 J. In comparison, the 0.8Si steel shows an ultimate tensile strength of 1029 MPa, an elongation after fracture of 50.5%, and an impact energy at 0 °C of 129.2 J, which is slightly lower than that of the 0Si steel. The addition of Si reduces the density of the experimental steel, and the 0.8Si steel exhibits a higher ultimate tensile strength; therefore, its specific strength (151.3 N·m/kg) is higher than that of the 0Si steel (147.9 N·m/kg).

### 3.2. Electrochemical Experimental Results

**Figure 2:**
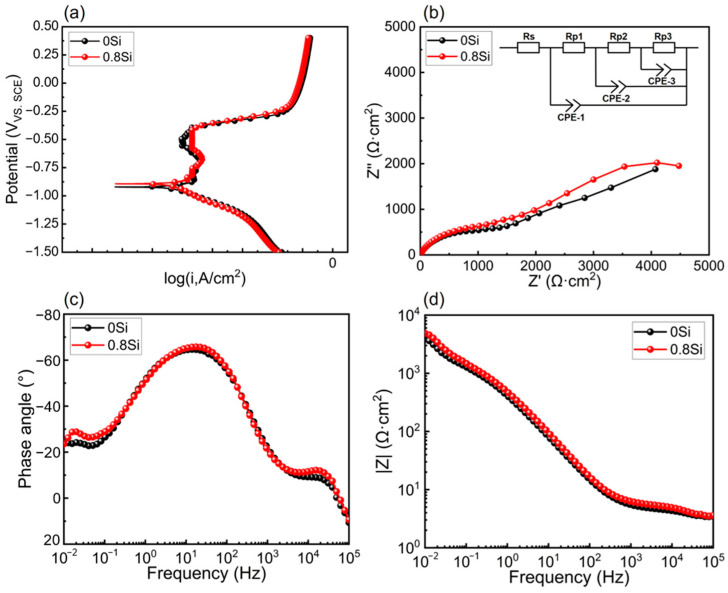
Electrochemical results for the experimental steel. (**a**) Potentiodynamic polarization curves; (**b**) impedance spectra and equivalent circuit; (**c**) phase angle plots; (**d**) impedance modulus plots.

The potentiodynamic polarization curves and electrochemical impedance spectroscopy (EIS) results (Nyquist and Bode plots) of the two experimental steels are shown in Figure 2. From the potentiodynamic polarization curves, the corrosion current density (icorr), corrosion potential (Ecorr), and pitting potential (Ep) of the two steels were determined, and the results are summarized in Table 3. As indicated by Figure 2a and Table 3, after the addition of Si, both Ecorr and Ep increase, while icorr and ip decreases, and a distinct passive region is observed for the experimental steels [27]. The passive current density of the 0.8Si steel is lower than that of the 0Si steel, indicating that the addition of Si to austenitic low-density steel affects both the intrinsic corrosion behavior and the characteristics of the passive film.

As shown in Figure 2b,c, the Nyquist and phase angle plots of the 0.8Si steel exhibit three arc segments, whereas the 0Si steel shows a straight line in the low-frequency region and arc-shaped features in the middle- and high-frequency regions. The arc in the high-frequency region corresponds to the charge-transfer process between the working electrode surface and the solution. Because an oxide film rapidly forms on the steel surface, the diameter of the high-frequency arc is extremely small and difficult to discern in the Nyquist plot; however, it can be clearly observed in the phase angle plot. The diameter of the high-frequency arc for the 0.8Si steel is larger than that for the 0Si steel, indicating a slower electron transfer rate between the electrode surface and the solution, which ultimately leads to the formation of a more uniform oxide film [28]. The arc in the medium-frequency region corresponds to the film impedance of the surface oxide layer [29,30,31], and the 0.8Si steel exhibits a larger arc radius, indicating a higher oxide film resistance. The low-frequency arc corresponds to the impedance of the corrosion product deposition layer formed after the gradual dissolution of the oxide film. The appearance of a low-frequency arc for the 0.8Si steel, in contrast to a straight line for the 0Si steel, indicates that the surface reaction at this stage is extremely rapid and no longer controlled by charge transfer but by ionic diffusion. This behavior demonstrates that the corrosion product deposition layer on the surface of the 0Si steel contains a higher density of pores, allowing a large number of solution ions to penetrate into the pores and react with the substrate [32].

### 3.3. Oxide Films Prepared by Potentiostatic Polarization

#### 3.3.1. Observation of Surface Oxide Film Morphology

**Figure 3:**
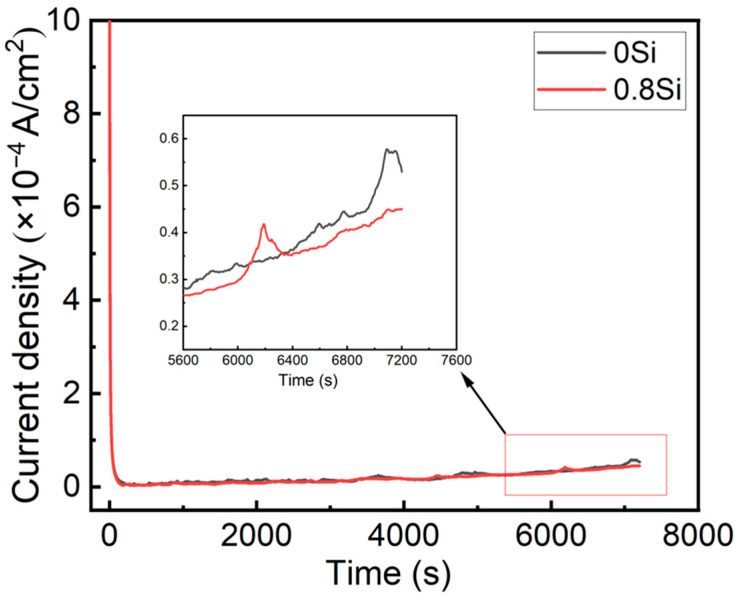
Potentiostatic polarization curves of experimental steel in 3.5 wt.% NaCl solution.

According to the electrochemical results, austenitic low-density steel initially forms a dense oxide film; subsequently, the oxide film dissolves or is locally broken down, followed by the formation of external corrosion products at later stages, ultimately leading to the development of a rust layer. Potentiostatic polarization tests were employed to evaluate the initial corrosion behavior of austenitic low-density steel and to prepare oxide films formed at the early stage of corrosion. Figure 3 shows the relationship between corrosion current density and time. Within the first 200 s of polarization, the current density of the experimental steels rapidly decreases to approximately 3 μA/cm^2^, corresponding to the formation of a surface oxide film and the initial anodic dissolution. This phenomenon indicates that austenitic low-density steel can rapidly form an oxide film within a short period, thereby suppressing anodic dissolution. With increasing polarization time, the current density gradually increases, indicating the progressive formation of corrosion products outside the oxide layer. The slight fluctuations observed in the polarization curves correspond to the breakdown and self-healing processes of the oxide film. Ultimately, the 0.8Si steel exhibits a lower steady-state current density, reflecting the higher compactness and relatively stronger protective capability of its oxide film.

**Figure 4:**
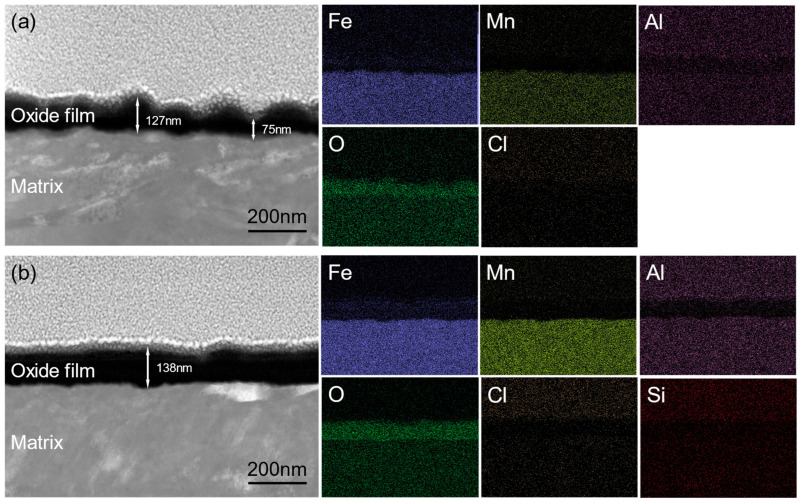
Morphology and elemental distribution of the experimental steel after polarization. (**a**) 0Si; (**b**) 0.8Si.

Cross-sectional transmission electron microscopy analyses were performed on the oxide films formed on the surfaces of the two experimental steels after polarization, and high-angle annular dark-field (HAADF) imaging together with scanning transmission electron microscopy energy-dispersive X-ray spectroscopy (STEM–EDS) mapping were obtained, as shown in Figure 4. The STEM–EDS results confirm the presence of oxygen in the films and reveal a uniform distribution of all alloying elements within the oxide layer [33]. From the morphological images, it can be clearly observed that the oxide film on the 0Si steel exhibits pronounced thickness inhomogeneity, with a large variation in thickness, ranging from approximately 75 nm at the thinnest region to about 127 nm at the thickest region. Such a highly nonuniform cross-sectional morphology directly reflects the presence of numerous structural defects in the oxide film on this steel, indicating relatively poor compactness of the film. The thinner regions of the oxide film represent weak points in resisting the attack of corrosive media; in Cl^−^ containing environments, these vulnerable sites are readily penetrated and disrupted by Cl^−^ ions, leading to the formation of pitting sites on the steel substrate and serving as initiation points for subsequent corrosion processes. In contrast, the oxide film formed on the surface of the 0.8Si steel exhibits not only a significantly increased thickness, reaching approximately 138 nm, but also a much more uniform thickness distribution, overall presenting a flat and compact cross-sectional morphology. This observation clearly demonstrates that the addition of Si to low-density steel effectively optimizes the growth process of the oxide film, promoting the formation of a protective layer with fewer defects and a more compact and stable structure. Such a dense and uniform oxide film can more effectively hinder the ingress of aggressive ions such as Cl^−^, substantially reducing the probability of ion penetration through the film and thereby exhibiting superior resistance to pitting corrosion during subsequent corrosion exposure.

#### 3.3.2. Phase Analysis of Surface Oxide Films

**Figure 5:**
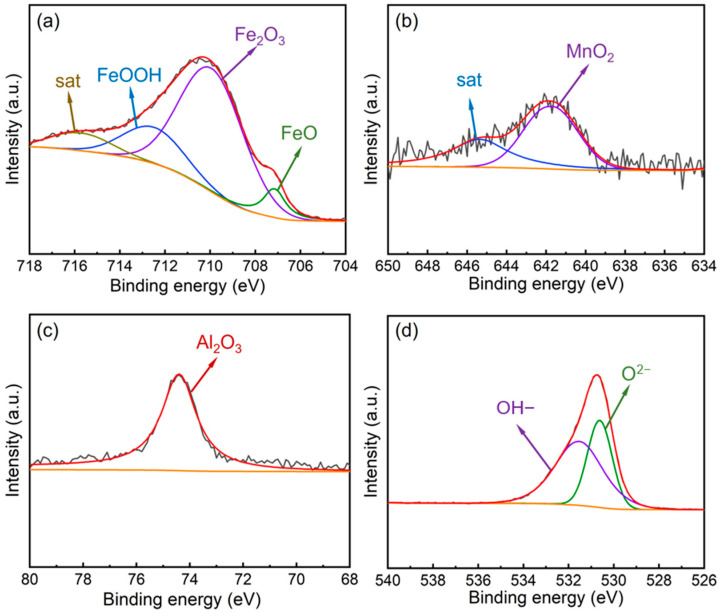
XPS spectra of the oxide film of 0Si experimental steel. (**a**) Fe 2p_3/2_; (**b**) Mn 2p_3/2_; (**c**) Al 2p; (**d**) O 1s.

To further determine the specific phase constituents of the oxide films and the relative contents of the constituent elements, X-ray photoelectron spectroscopy (XPS) analyses were conducted on the surface oxide films formed on the two experimental steels after polarization. Figure 5 presents the XPS spectra of Fe 2p_3/2_, Mn 2p_3/2_, Al 2p, and O 1s for the oxide film on the 0Si steel. According to the Fe 2p_3/2_ spectrum of the oxide film on the 0Si steel (Figure 5a), Fe is present in the forms of FeO (707.0 eV), Fe_2_O_3_ (710.2 eV), FeOOH (712.7 eV), and a satellite peak (715.9 eV) [34]. These results indicate that Fe has undergone further corrosion; not only has an oxide film formed on the steel surface, but a considerable amount of Fe has also diffused outward and been further corroded outside the oxide film, suggesting that the oxide film formed on the 0Si steel is insufficiently compact to effectively inhibit the outward diffusion of substrate elements. Figure 5b shows the Mn 2p_3/2_ XPS spectrum of the oxide film on the 0Si steel, which is mainly composed of MnO_2_ (641.8 eV), with the satellite peak at 646.2 eV corresponding to Mn(OH)_2_. The Al 2p spectrum (Figure 5c) exhibits a single peak attributed to Al_2_O_3_ (74.3 eV). According to the O 1s spectrum (Figure 5d), oxygen in the surface oxide film exists in the forms of O^2−^ (530.6 eV) and OH^−^ (531.6 eV), corresponding to oxides and hydroxides, respectively.

**Figure 6:**
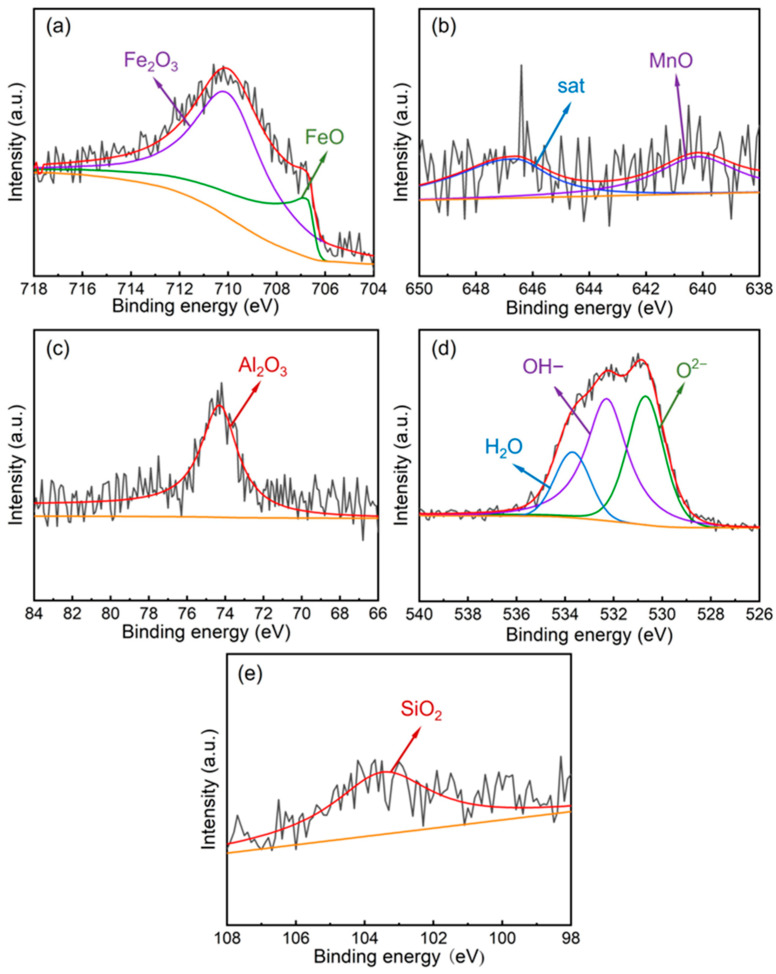
XPS spectra of the oxide film of 0.8Si experimental steel. (**a**) Fe 2p_3/2_; (**b**) Mn 2p_3/2_; (**c**) Al 2p; (**d**) O 1s (**e**) Si 2p.

According to the Fe 2p_3/2_ spectrum of the oxide film on the 0.8Si steel (Figure 6a), Fe is present only in the forms of FeO (707.0 eV) and Fe_2_O_3_ (710.2 eV), with no FeOOH peak detected, indicating that only an oxide film is formed on the steel surface with good compactness, which effectively prevents the outward diffusion of substrate elements. Figure 6b shows the Mn 2p_3/2_ XPS spectrum of the oxide film on the 0.8Si steel, which is mainly composed of MnO (640.2 eV) and satellite peaks. The Al 2p spectrum (Figure 6c) also exhibits a single peak corresponding to Al_2_O_3_ (74.3 eV). The Si 2p spectrum (Figure 6d) presents a single peak attributed to SiO_2_ (103.4 eV), demonstrating that the Si added to austenitic low-density steel participates in the formation of the oxide film at the initial stage of corrosion. The formed SiO_2_ is amorphous; compared with the crystalline structures of other oxides, the amorphous phase can more effectively bond with the substrate and surrounding crystalline phases, thereby enhancing the compactness and stability of the oxide film. The O 1s spectrum (Figure 6e) consists of peaks corresponding to O^2−^ (530.6 eV), OH^−^ (531.6 eV), and H_2_O (533.7 eV), which are associated with oxides, hydroxides, and crystallization water, respectively.

### 3.4. Results of the Periodic Immersion Accelerated Corrosion Test

#### 3.4.1. Measurement of Corrosion Weight Loss and Calculation of Corrosion Rate

**Figure 7:**
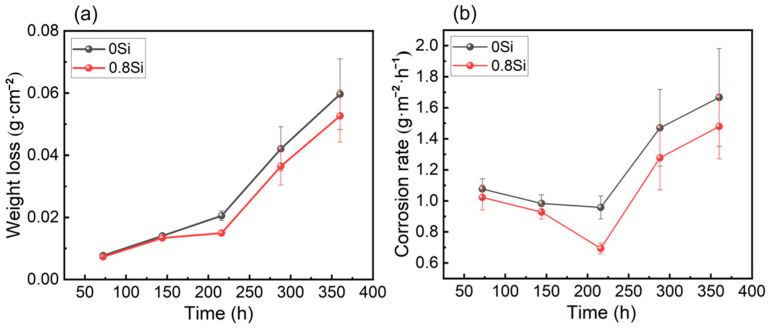
Mass loss and corrosion rate of experimental steel in different exposure time. (**a**) Weight loss; (**b**) corrosion rate.

After the periodic immersion accelerated corrosion tests in a 3.5 wt.% NaCl solution, the mass loss and corrosion rate variations in the two experimental steels at different corrosion durations are shown in Figure 7. As illustrated in Figure 7a, the mass loss of the experimental steels increases progressively with increasing corrosion duration, and at each corrosion interval, the mass loss of the 0.8Si steel is significantly lower than that of the 0Si steel. Based on the mass loss data of the experimental steels at different corrosion durations, the corrosion rates were calculated using Equation (1).

(1) $$v=\frac{∆m}{t\times S}$$

where *v* is the corrosion rate (g·m^−2^·h^−1^); Δ*m* is the mass loss of the experimental steel before and after corrosion (g); *t* is the corrosion time (h); and *S* is the surface area of the experimental steel (m^2^). Figure 7b shows the variation in corrosion rate of the experimental steels at different corrosion durations, exhibiting a trend of an initial decrease followed by an increase. This behavior can be attributed to the rapid formation of a dense oxide film on the steel surface at the early stage of corrosion, which effectively inhibits the outward diffusion of metal atoms from the substrate. The oxide film formed on the 0.8Si steel provides superior protection compared with that on the 0Si steel; therefore, during the period from 72 to 216 h, the corrosion rates of both steels decrease, with a more pronounced decline observed for the 0.8Si steel. When corrosion proceeds for a sufficiently long duration, rust layer spallation occurs, and the oxide film has largely dissolved. Consequently, aggressive Cl^−^ ions from the environment gradually penetrate the surface, and metal elements from the substrate diffuse outward and react with oxygen, leading to an increase in the corrosion rate.

#### 3.4.2. Morphology and Elemental Distribution of Corrosion Products

**Figure 8:**
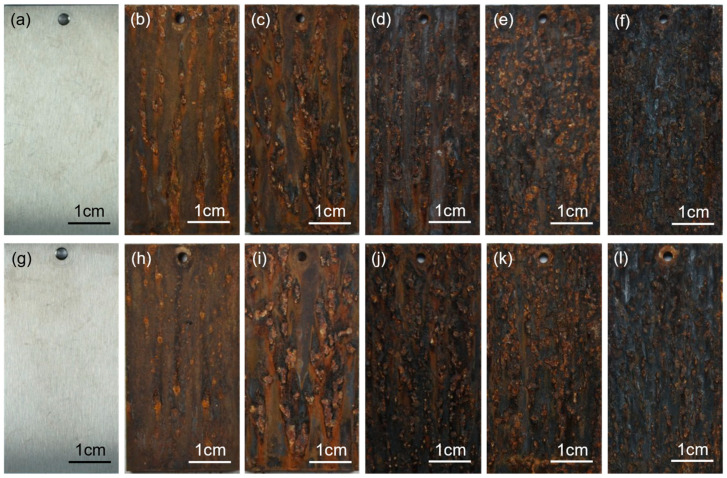
Macroscopic morphology of experimental steel in different corrosion times. (**a**) 0 h–0Si; (**b**) 72 h–0Si; (**c**) 144 h–0Si; (**d**) 216 h–0Si; (**e**) 288 h–0Si; (**f**) 360 h–0Si; (**g**) 72 h–0Si; (**h**) 72 h–0.8Si; (**i**) 144 h–0.8Si; (**j**) 216 h–0.8Si; (**k**) 288 h–0.8Si; (**l**) 360 h–0.8Si.

Figure 8 shows the macroscopic morphologies of the two experimental steels after periodic immersion accelerated corrosion tests at different corrosion durations. After 72 h of periodic immersion, rust layers formed on the specimen surfaces, which were mainly yellow–brown in color, and corrosion pits were clearly observed; the number of corrosion pits on the 0.8Si steel was significantly lower than that on the 0Si steel. As the corrosion duration increased to 144 h, part of the rust layer began to transform into a brown–black color. After 216 h of exposure in the saline environment, most of the rust layer had transformed into brown–black rust; however, partial rust spallation occurred in the smooth, pit-free regions on the surface of the 0Si steel, exposing the substrate, whereas such spallation was not evident on the 0.8Si steel. Due to rust layer spallation and exposure of the substrate, corrosion was re-initiated at these locations; therefore, after 288 h, the steel surfaces exhibited newly formed yellow–brown rust layers, corresponding to corrosion products generated over a relatively short exposure time. After 360 h of exposure in the saline environment, owing to the prolonged exposure duration, almost the entire surface was transformed into brown–black rust layers.

**Figure 9:**
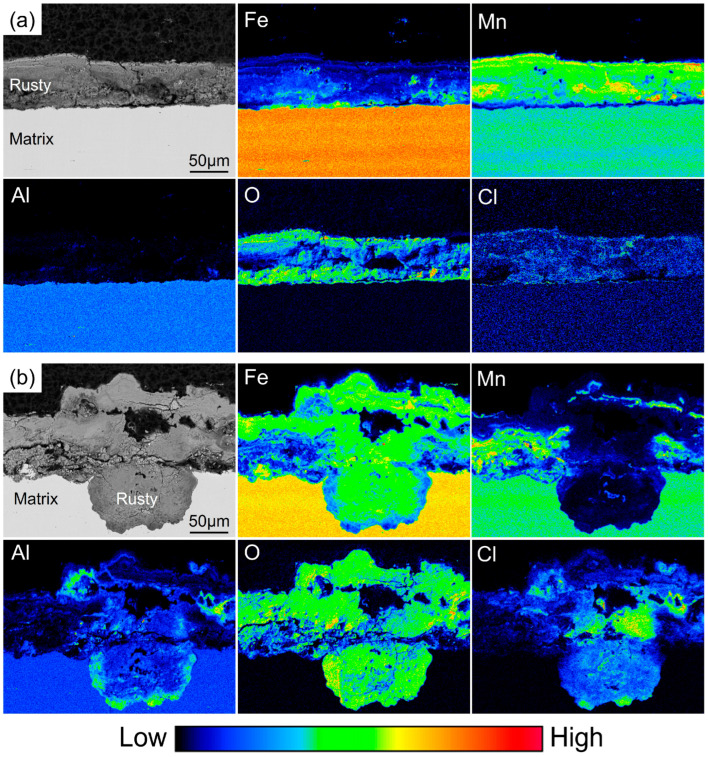
Elemental distribution of corrosion product cross-section in 0Si steel. (**a**) Flat region; (**b**) pitting region.

Because the Si content added to the low-density steel is relatively low, electron probe microanalysis (EPMA) was employed to accurately determine the distribution of Si within the rust layer by analyzing the elemental distribution across the rust-layer cross section. Based on the corrosion rate curves and macroscopic observations of the rust layers, relatively uniform corrosion products were formed after 360 h of periodic immersion, indicating that the corrosion process had entered a later stage; therefore, specimens subjected to 360 h of periodic immersion were selected for EPMA analysis. EPMA can qualitatively characterize the occurrence state of elements in the rust layer and the substrate. The color scale from blue to red represents the relative content of elements from low to high. By comparing the contrast of various colors in the figure can roughly determine where the elements are enriched. Figure 9 shows the elemental distributions in the rust layers of the 0Si steel after 360 h of periodic immersion, including both flat regions and pitting areas. It can be observed that, in the flat regions, the rust layer is mainly composed of Mn corrosion products, and an Fe-enriched zone is present at the interface between the rust layer and the substrate. The Al content in the rust layer is very low, with almost no Al-enriched regions, while the corrosive element Cl is relatively uniformly distributed throughout the entire rust layer. In contrast, in the pitting regions, there is almost no Mn enrichment inside the pits; therefore, the rust layer in these areas is considered to be mainly composed of corrosion products of Fe and Al. Moreover, a pronounced Al-enriched zone is observed near the substrate within the pits, and a large amount of Cl enrichment is also detected inside the pits.

**Figure 10:**
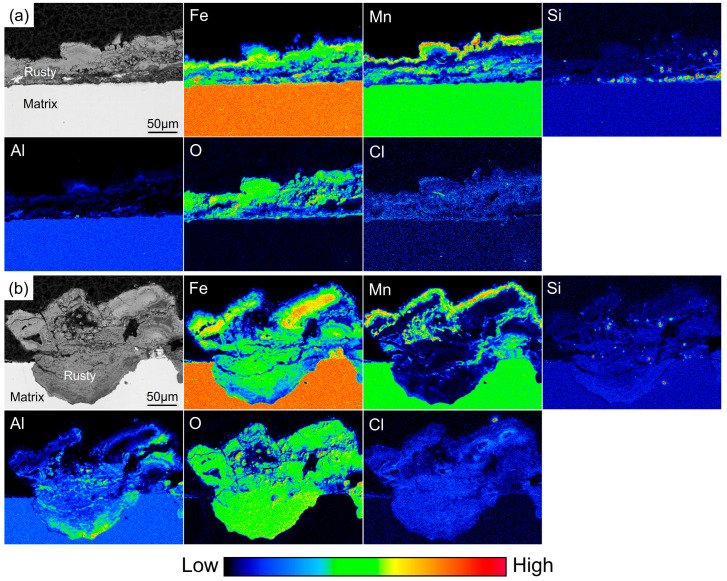
Elemental distribution of corrosion product cross-section in 0.8Si steel. (**a**) Flat region; (**b**) pitting region.

Figure 10 shows the elemental distributions in the rust layers of the 0.8Si steel after 360 h of periodic immersion, including both flat regions and pitting regions. In the flat regions, the rust layer is mainly composed of corrosion products of Mn and Fe; Fe is distributed within the interior of the rust layer, while Mn is heavily enriched in the outermost layer. Al-enriched zones are observed in both the outermost and innermost layers of the rust layer. O and Cl remain relatively uniformly distributed throughout the rust layer; however, a small amount of Cl enrichment is also detected at the Al-enriched positions in the outermost layer, indicating that Al plays a more pronounced role in hindering the inward diffusion of Cl^−^ within the rust layer. A significant enrichment of Si is observed at the interface between the rust layer and the substrate, and this enrichment largely coincides with the Al-enriched regions within the rust layer. Combined with the XPS results, these Al- and Si-enriched regions are inferred to correspond to the surface oxide film that has just been dissolved. In the pitting regions, the rust layer inside the pits is still composed of corrosion products of Fe and Al, while Mn is distributed in the outermost layer of the entire rust layer. Si is also detected inside the pits, with its enrichment becoming more pronounced closer to the substrate, and Al remains enriched near the substrate.

#### 3.4.3. Phase Analysis of Corrosion Products

**Figure 11:**
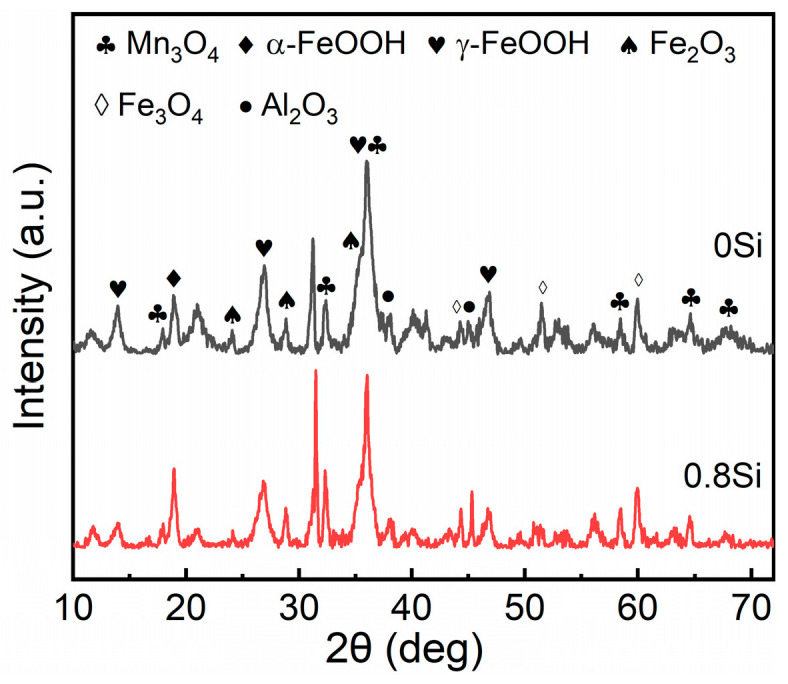
X-ray diffraction analysis of two steels after periodic immersion in 360 h.

The differences in rust-layer morphology are essentially a macroscopic manifestation of variations in the phase composition of the corrosion products. To further determine the effect of Si on the corrosion products formed on the experimental steels after the periodic immersion, rust powders were collected from the steel surfaces and characterized by X-ray diffraction (XRD) to identify the rust-layer constituents and to perform semi-quantitative analysis of their contents. Figure 11 compares the main constituents of the rust layers formed on the experimental steels after 360 h of periodic immersion. Analysis of the XRD peaks indicates that the primary components of the rust layers are Mn_3_O_4_, α-FeOOH, γ-FeOOH, Fe_2_O_3_, Fe_3_O_4_, and Al_2_O_3_. After the addition of Si, the intensity and width of the diffraction peaks of some corrosion products change, indicating that Si influences the formation of certain oxides within the rust layer. No diffraction peaks associated with Si oxides are detected in the spectrum of the 0.8Si steel, which is likely due to their low content being below the detection limit of XRD [35].

**Figure 12:**
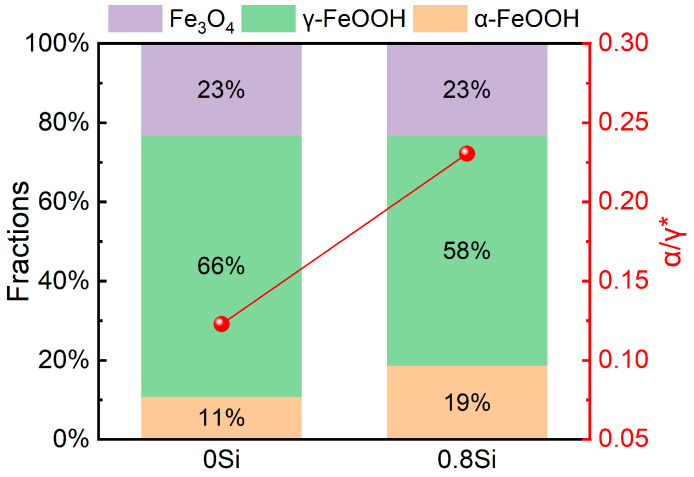
Proportion of each phase in rust layer of two steels after periodic immersion in 360 h. The red dots represent the α*/γ ratio in samples with different Si contents.

Among the corrosion products of low-density steel, various iron oxides and oxyhydroxides remain the dominant constituents. Among them, γ-FeOOH is a metastable phase that can subsequently transform into the structurally stable α-FeOOH. The presence of Fe_3_O_4_ leads to an inhomogeneous distribution of corrosion products within the rust layer, and its porous nature prevents effective blockage of corrosive media ingress, thereby resulting in an increased corrosion rate at later stages. Therefore, to elucidate the effect of Si on the compactness of the rust layer in low-density steel and to provide an intuitive explanation of the influence of Si on the amount of corrosion products formed, a semi-quantitative calculation of the α/γ* ratio was employed, where α represents α-FeOOH and γ* denotes the sum of γ-FeOOH and Fe_3_O_4_; the results are shown in Figure 12. After 360 h of exposure in the saline environment, the mass fraction of Fe_3_O_4_ in the rust layers of both experimental steels is identical, at 23%, while the mass fractions of γ-FeOOH are 66% and 58%, and those of α-FeOOH are 11% and 19%, respectively. After 360 h of periodic immersion, the rust layer of the 0.8Si steel exhibits a lower mass fraction of γ-FeOOH and a higher mass fraction of α-FeOOH, and the calculated α/γ* value is higher than that of the 0Si steel. These results indicate that the addition of Si promotes the structural transformation of γ-FeOOH to α-FeOOH, increases the content of corrosion-resistant phases in the rust layer, and thereby enhances the compactness and stability of the rust layer, providing more effective protection for the substrate.

#### 3.4.4. Surface Morphology of Experimental Steels After Rust Removal

**Figure 13:**
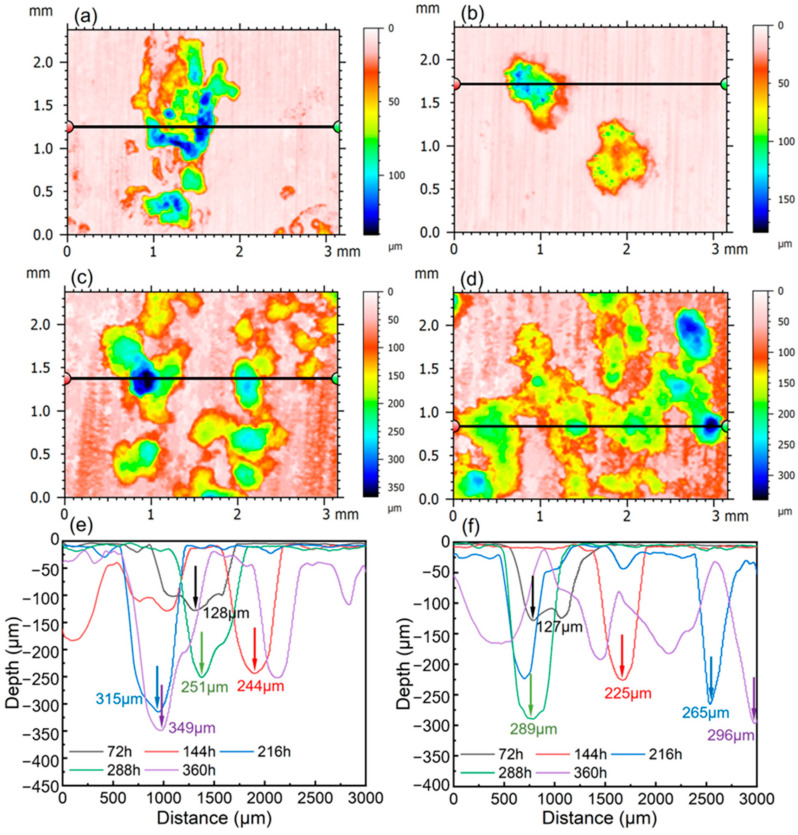
Surface morphology and maximum pit depth of two experimental steels. (**a**) Surface morphology of 0Si steel after 72 h; (**b**) Surface morphology of 0.8Si steel after 72 h; (**c**) Surface morphology of 0Si steel after 360 h; (**d**) Surface morphology of 0.8Si steel after 360 h; The maximum pit depth was measured along the horizontal line passing through the deepest pit point in (**a**–**d**). (**e**) Maximum pit depth of 0Si steel; (**f**) maximum pit depth of 0.8Si steel.

Due to the high Al content in the matrix of low-density steel, a dense surface oxide film forms rapidly during corrosion in a marine environment, resulting in pronounced passivation behavior. As corrosion progresses, defect sites in the oxide film are broken down by Cl^−^, leading to the formation of pitting pits. These pits propagate into the depth of the material and gradually become crack initiation sites; the cracks continue to grow, ultimately resulting in material failure. Figure 13 shows the surface morphologies of the derusted specimens of the two experimental steels after periodic immersion, as captured by a three-dimensional laser confocal microscope. Overall, regardless of whether the corrosion duration is short (72 h) or long (360 h), the pits formed on the 0.8Si steel are smaller and shallower than those on the 0Si steel, which is consistent with the superior compactness of the oxide film on the 0.8Si steel. The maximum pit depths on the surfaces of the two experimental steels are shown in Figure 13e,f. After 72 h of periodic immersion, the pit depths of the two steels are similar, at 128 μm and 129 μm, respectively; however, the number and size of pits on the 0Si steel are significantly greater than those on the 0.8Si steel. This indicates that the oxide films initially formed on both steels can be broken down by Cl^−^, but the 0Si steel possesses more defect sites and a less compact oxide film, resulting in a greater number of pit initiation sites. After 360 h of periodic immersion, the maximum pit depth on the surface of the 0.8Si steel is 296 μm, which is markedly lower than that of the 0Si steel (349 μm), indicating that the growth rate of pit depth in the 0Si steel is significantly faster than that in the 0.8Si steel. This finding demonstrates that the addition of Si not only enhances the compactness of the oxide film formed at the initial stage of corrosion in low-density steel, but also facilitates its timely reconstruction after breakdown, thereby hindering further deepening of pitting pits. The EPMA results reveal pronounced enrichment of Al and Si at the bottoms of the pits, which is consistent with this conclusion.

## 4. Discussion

### 4.1. Elemental Distribution in Oxide Films

**Figure 14:**
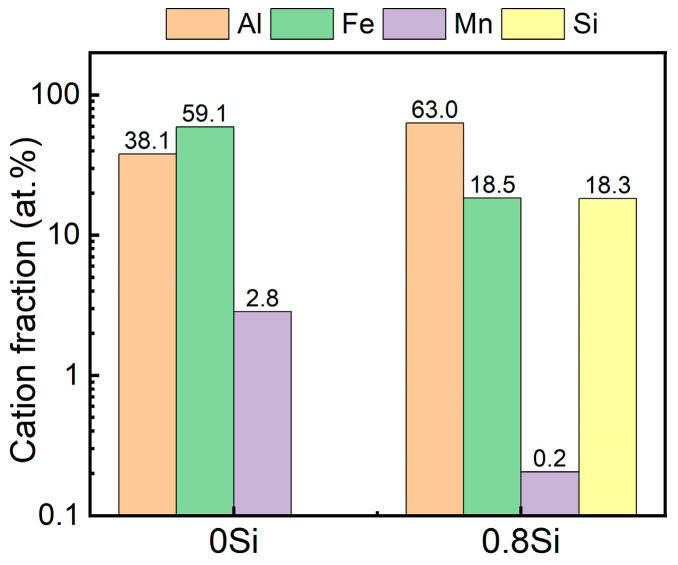
Variation in each element in the oxide film of the experimental steel.

The relative contents of the elements in the oxide films of the two experimental steels were calculated using Equation (2) [36], and the results are presented in Figure 14.

(2) $$C_{X}=\frac{I_{X}/S_{X}}{\sum I_{i}/S_{i}}$$

where *I_X_* is the peak area of the corresponding element and *S_X_* is the XPS sensitivity factor. Because low-density steel contains a relatively high Al content, Al is rapidly oxidized at the early stage of corrosion to form a dense alumina film; therefore, the dominant metallic element in the oxide film is expected to be Al. However, Fe is identified as the major metallic element in the oxide film of the 0Si steel, indicating that additional corrosion products have already formed outside the oxide film, which indirectly demonstrates the poor compactness of the oxide film. In contrast, Al is the dominant metallic element in the oxide film of the 0.8Si steel, confirming that only a dense oxide film is formed on the sample surface at this stage. In the 0.8Si steel, the relative contents of Si and Fe are approximately comparable; however, since the Si content in the steel matrix is much lower than that of Fe, it can be inferred that, during the initial stage of corrosion in a marine environment, Si reacts with oxygen more readily than Fe to form oxides. Both experimental steels exhibit the lowest Mn content in the oxide films, with the Mn content being even lower in the 0.8Si steel. During the initial corrosion process of austenitic low-density steel, Mn tends to form oxides with poor compactness, which deteriorate the compactness of the oxide film; therefore, this finding demonstrates that the addition of Si not only participates in oxidation to form SiO_2_ but also significantly suppresses the oxidation of Mn and promotes the oxidation of Al, thereby enhancing the compactness of the oxide film. Comparable Al- and Si-enriched surface films in chloride-containing solutions have been reported for Fe-Mn-Al-Si steels, and these studies consistently show that the corrosion performance is highly sensitive to the balance between Al/Si-derived oxides and Mn-derived oxides/hydroxides in the surface film [37]. In particular, Mn enrichment in the outer oxide layer (e.g., MnO/MnO_2_) has been associated with a less protective film and increased corrosion susceptibility, whereas Al_2_O_3_ and Si-containing ox-ides/hydroxides contribute to a denser and more stable surface layer [20].

### 4.2. Thermodynamic Analysis of Surface Oxide Film Formation

The oxide film formed at the initial stage of corrosion plays a crucial role in determining the corrosion resistance of austenitic low-density steel. In the oxide film, Al is the dominant metallic element, while Si also undergoes rapid oxidation and forms SiO_2_ within the oxide film. The standard Gibbs free energy (ΔG°) values of the oxidation reactions of Al, Si, Fe, and Mn at room temperature are listed in Table 4. According to the Ellingham diagram, a lower standard Gibbs free energy corresponds to a stronger tendency for oxidation. The oxidation curve of Al consistently appears at the bottom of the Ellingham diagram, indicating that Al preferentially oxidizes to form thermodynamically stable Al_2_O_3_ even under oxygen-deficient conditions. The oxidation curve of Si lies only slightly above that of Al in the Ellingham diagram and thus Si is also readily oxidized [38]; however, due to its very low content, the XPS results of the 0.8Si steel shown in Figure 14 indicate that, at the early stage of corrosion, the content of SiO_2_ in the oxide film is lower than that of Al_2_O_3_. In contrast, the oxidation reactions of Fe and Mn exhibit higher ΔG° values, making them less prone to oxidation under oxygen-deficient conditions [39]; therefore, relatively lower fractions of Fe and Mn are observed in the phase analysis of the oxide film. From an electrochemical perspective, the standard electrode potentials (E°) of these four elements are listed in Table 5. The more negative the standard electrode potential, the stronger the tendency of a metal to undergo oxidation. Al exhibits the most negative potential, demonstrating its strongest tendency for electron loss and anodic dissolution, which provides the electrochemical driving force for its oxidation process. Therefore, the present thermodynamic analysis should be interpreted together with kinetic and transport effects: in NaCl solutions, selective oxidation, competitive dissolution, and element redistribution can lead to an outer Mn-rich, defective layer and an inner Al-rich layer, as reported by XPS and EIS studies on high-Mn austenitic Fe-Mn-Al(-Si) steels. This framework is consistent with Figure 14, where Si addition reduces Mn incorporation into the oxide film while maintaining an Al-dominant film chemistry, which is expected to increase film resistance and retard chloride-assisted breakdown [37].

### 4.3. Transformation Mechanism of Corrosion Products

During the corrosion process of low-density steel, an oxide film preferentially forms on the substrate surface. As the exposure time increases, the oxide film gradually dissolves, substrate elements progressively diffuse outward, and a brown–black rust layer, as shown in Figure 8, is ultimately formed. Because Al has the most negative standard Gibbs free energy for oxide formation, it exhibits the fastest outward diffusion rate and therefore appears in the outermost layer of the rust layer, followed by Mn, while Fe is mainly distributed in the innermost layer. However, no Al is observed in the rust layer of the 0Si steel shown in Figure 9, whereas partial Al enrichment is detected in the outermost layer of the rust layer of the 0.8Si steel shown in Figure 10. This indicates that Al is indeed distributed in the outermost rust layer but tends to combine with Cl^−^ and subsequently spall off, which also explains why relatively low Cl enrichment is observed in the rust layers at the flat regions in Figure 9 and Figure 10. In the innermost region of the flat rust layer of the 0.8Si steel, Al- and Si-enriched zones are detected, which are considered to correspond to the oxide film that has just been dissolved. This observation demonstrates that the addition of Si enhances the stability of Al_2_O_3_, resulting in a more compact oxide film that is less prone to dissolution compared with that formed on the 0Si steel. In addition to dissolution, the oxide film can also be broken down by Cl^−^ ions, leading to the formation of pitting pits; the enrichment of Al and Si at the bottoms of the pits indicates that the oxide film undergoes reconstruction after breakdown, thereby hindering further deepening of the pits. Consequently, as shown in Figure 13, the 0.8Si steel exhibits a lower pitting depth. Similar synergistic effects between Al- and Si-containing oxides/hydroxides have been reported for Fe-Mn-Al-Si-C alloys in NaCl media, where hydrated alumina and amor-phous silica species form a relatively protective scale, while Mn-rich compounds are generally less compact [40].

Studies have shown that the chemical composition and compactness of the rust layer determine its ability to inhibit corrosion of the substrate, and that the relative proportions of oxidation products such as α-FeOOH, β-FeOOH, γ-FeOOH, Fe_3_O_4_, and Fe_2_O_3_ can differ significantly under identical service conditions [41]. Among the iron oxyhydroxides present in rust layers, the corrosion resistance increases in the order β-FeOOH < γ-FeOOH < α-FeOOH. In marine environments containing high concentrations of Cl^−^, large amounts of β-FeOOH are generally formed [42]. β-FeOOH possesses a relatively loose structure, is prone to moisture absorption, and exhibits strong chloride-ion adsorption capability and oxidizing activity. However, β-FeOOH is not detected in the XRD patterns shown in Figure 11. This is because β-FeOOH, as a highly reactive and readily reducible intermediate product, although it forms in large quantities within the rust layer, has largely transformed into more stable oxides such as Fe_3_O_4_ and α-FeOOH after 360 h of periodic immersion, and therefore its diffraction peaks are not observed in XRD measurements [43]. The reduction priority of γ-FeOOH is lower than that of β-FeOOH; nevertheless, this metastable phase ultimately transforms into the structurally stable α-FeOOH. α-FeOOH has an orthorhombic crystal structure, and owing to its high chemical stability, it is generally regarded as a protective corrosion product in rust layers. Under specific environmental conditions, α-FeOOH can effectively inhibit further corrosion of the substrate [44], thereby slowing the corrosion rate of low-density steel and providing protection to the substrate. In addition, immersion and polarization studies on Fe-Mn-Al-Si steels in 3.5% NaCl indicate that corrosion tends to be localized and strongly governed by passive-film compactness and its resistance to chloride-induced breakdown, supporting the emphasis on film integrity and reconstruction in the present work.

## 5. Conclusions

Electrochemical experiments conducted in a 3.5 wt.% NaCl solution yielded the potentiodynamic polarization curves and electrochemical impedance spectra of the two experimental steels. Compared with the 0Si steel (E_corr_ = −0.93 ± 0.024 V_vs.SCE_, I_corr_ = 4.28 ± 1.233 μA/cm^2^, E_p_ = −0.48 ± 0.018 V_vs.SCE_, I_p_ = 27.79 ± 2.563 μA/cm^2^), the 0.8Si steel shows a nobler E_corr_ (−0.90 ± 0.020 V_vs.SCE_) and higher E_p_ (−0.38 ± 0.016 V_vs.SCE_) together with reduced I_corr_ (3.84 ± 0.985 μA/cm^2^) and I_p_ (20.94 ± 3.070 μA/cm^2^). Mean-while, the 0.8Si steel exhibits a larger impedance arc radius with three time-constant features, indicating higher charge-transfer/film resistance and thus superior corrosion resistance.

Potentiostatic polarization tests performed on both experimental steels resulted in the formation of dense oxide films on the surfaces. Within the first ~200 s of polarization, the current density rapidly decreased to ~3 μA/cm^2^, and the 0.8Si steel reached a lower steady-state current density after 7200 s at −0.5 V_SCE_. Cross-sectional TEM reveals that the oxide film on the 0Si steel is highly nonuniform (≈75–127 nm), whereas the 0.8Si steel forms a thicker and much more uniform film (≈138 nm). Consistently, XPS shows FeOOH is detected in the 0Si film but is absent in the 0.8Si film, while Si participates in film formation as SiO_2_ (Si 2p at 103.4 eV) together with Al_2_O_3_ (Al 2p at 74.3 eV), demonstrating that Si addition improves oxide-film compactness/uniformity, enhances substrate protection, and reduces pitting initiation sites.

Periodic immersion accelerated corrosion tests on the two low-density steels reveal that the corrosion rate is significantly reduced after the addition of Si throughout 72–360 h exposure. After 360 h, XRD semi-quantification indicates identical Fe_3_O_4_ mass fractions (23%) for both steels, but γ-FeOOH decreases from 66% (0Si) to 58% (0.8Si) while α-FeOOH increases from 11% to 19%, resulting in a higher α/γ* ratio and a more stable/compact rust layer. EPMA analysis shows pronounced Al and Si enrichment in the inner rust layer and at pitting cavities, and 3D confocal measurements further con-firm suppressed pit growth: the maximum pit depth is reduced from 349 μm (0Si) to 296 μm (0.8Si) after 360 h (72 h: 128 μm vs. 129 μm). Together, these results demonstrate that Si effectively inhibits the initiation and propagation of pitting corrosion by promoting protective film/rust-layer evolution.

## Figures and Tables

**Table 1:** Chemical composition of experimental steel (wt.%).

wt.%	C	Si	Mn	Al	Fe
0Si	0.9	0	29.0	7.5	Bal.
0.8Si	0.9	0.8	29.0	7.5	Bal.

**Table 2:** Mechanical properties of experimental steel.

Steel	Rp_0.2_ (MPa)	R_m_ (MPa)	A_50_ (%)	A_g_ (%)	KV_2_ (0 °C) (J)	Rm/ρ (N·m/kg)
0Si	697	1010	51.9	41.4	148.2	147.9
0.8Si	751	1029	50.5	40.4	129.2	151.3

**Table 3:** The electrochemical parameters of experimental steel.

Steel	Ecorr (V_vs.SCE_)	Icorr (μA/cm^2^)	Ep/ (V_vs.SCE_)	Ip/ (μA/cm^2^)
0Si	−0.93 ± 0.024	4.28 ± 1.233	−0.48 ± 0.018	27.79 ± 2.563
0.8Si	−0.90 ± 0.020	3.84 ± 0.985	−0.38 ± 0.016	20.94 ± 3.070

**Table 4:** Standard Gibbs free energy of major alloying elements at room temperature.

	Al → Al_2_O_3_	Fe → FeO	Mn → MnO	Si → SiO_2_
ΔG° (kJ/mol)	−756.3	−116.7	−173.4	−856.6

**Table 5:** Standard electrode potentials of major alloying elements at room temperature.

	Al^3+^/Al	Mn^2+^/Mn	Fe^2+^/Fe	SiO_2_/Si
E° (V_vs.SCE_)	−1.66	−1.18	−0.44	−0.86

## Data Availability

The original contributions presented in this study are included in the article. Further inquiries can be directed to the corresponding author.

## References

[1] Shojai S., Schaumann P., Braun M., Ehlers S. (2022). Influence of pitting corrosion on the fatigue strength of offshore steel structures based on 3D surface scans. Int. J. Fatigue.

[2] Zanuttigh B., Dallavalle E., Zagonari F. (2025). A novel framework for sustainable decision-making on reusing Oil & Gas offshore platforms with application to the Adriatic Sea. Renew. Sustain. Energy Rev..

[3] Karimi H.R., Shabakhty N. (2023). A comparative assessment of the impact of tilting on structural safety and behavior of fixed offshore platforms. Appl. Ocean Res..

[4] Edwards E.C., Holcombe A., Brown S., Ransley E., Hann M., Greaves D. (2024). Trends in floating offshore wind platforms: A review of early-stage devices. Renew. Sustain. Energy Rev..

[5] Ben N., Vytyaz O.Y., Hrabovskyy R.S. (2023). Mechanical Properties of Steel for Floating Offshore Platforms Under Static and Cyclic Loading. Mater. Sci..

[6] Mimica D., Skejic D., Catipovic I., Parunov J. Framework for the Lifetime Extension of Fixed Offshore Platforms in the Northern Adriatic Sea. Proceedings of the 26th Symposium on the Theory and Practice of Shipbuilding-SORTA.

[7] Shojai S., Schaumann P., Bromer T. (2022). Probabilistic modelling of pitting corrosion and its impact on stress concentrations in steel structures in the offshore wind energy. Mar. Struct..

[8] Liu C., Xu Q., Liu X., Fan X., Li Y. (2024). Comparative study of marine steel and welding joint in artificial seawater based on stress corrosion cracking and crack growth. J. Mater. Res. Technol..

[9] Amin Z., Hamed K., Afshin R. (2022). Fault-tolerant control for nonlinear offshore steel jacket platforms based on reinforcement learning. Ocean Eng..

[10] Ji H., Wang H., Chen Q., Ma X., Cai Y. (2024). Corrosion behavior prediction for hull steels under dynamic marine environments by jointly utilizing LSTM network and PSO-RF model. Ocean Eng..

[11] Lu Y., Liu Z.X., Wang P.P., Wang H.Y. (2025). Corrosion failure mechanism and electrolytic accelerated fatigue performance of butt-welded joints. J. Constr. Steel Res..

[12] Liu H.X., Gao H.M., Chen J.M., Liu R.L., Zhang Y., Yin Y.S., Liu H.F., Fan S.J., Liu H.W. (2025). Study of corrosion and failure mechanism of the galvanized steel pipes of meteorological tower serviced in the South China Sea. Eng. Fail. Anal..

[13] Du J., Chen P., Zhang F., Jia Z., Shi F., Li X. (2025). Controllable κ-carbide precipitation enables strength-ductility co-enhancement in Fe-Mn-Al-C low-density austenitic steel via grain boundary engineering. J. Mater. Sci. Technol..

[14] Wang S.T., Sun Z.D., Li D.J., Yu Q., Wang Q.F. (2025). Effect of Aluminum Content on the Corrosion Behavior of Fe-Mn-Al-C Structural Steels in Marine Environments. Metals.

[15] Gutierrez-Urrutia I. (2021). Low Density Fe-Mn-Al-C Steels: Phase Structures, Mechanisms and Properties. ISIJ Int..

[16] Zhao L., van Dijk N.H., Brück E., Sietsma J., van der Zwaag S. (2001). Magnetic and X-ray diffraction measurements for the determination of retained austenite in TRIP steels. Mater. Sci. Eng. A-Struct. Mater. Prop. Microstruct. Process..

[17] Bhattacharya B., Sharma A.S., Hazra S.S., Ray R.K. (2009). A Study of Microstructures and Tensile Properties of Two Fe-Mn-Al-Si-C Alloys. Metall. Mater. Trans. A-Phys. Metall. Mater. Sci..

[18] Jia Q.J., Jiang X.Q., Wu C.J., Chen J.X., Zhu X.Y., Liu Y., Su X.P. (2025). Effect of Si on Mechanical Properties and Oxide Film Formation of AFA Alloy at Low Oxygen Pressure. Coatings.

[19] Heon-Young H., Kyeong-Won K., Seong-Jun P., Tae-Ho L., Hyungkwon P., Joonoh M., Hyun-Uk H., Chang-Hoon L. (2022). Effects of Cr on pitting corrosion resistance and passive film properties of austenitic Fe–19Mn–12Al–1.5 C lightweight steel. Corros. Sci..

[20] Bosch J., Martin U., Aperador W., Bastidas J.M., Ress J., Bastidas D.M. (2021). Corrosion Behavior of High-Mn Austenitic Fe–Mn–Al–Cr–C Steels in NaCl and NaOH Solutions. Materials.

[21] Jinbin Z., Wei W., Jiaxing C., Xuequn C. (2022). Evaluating the effect of aluminum on the corrosion resistance of the structural steels used for marine engineering. J. Mater. Res. Technol..

[22] Haixia L., Feng H., Wei Y., Qian H., Jing L., Frank C.Y. (2020). Essential role of element Si in corrosion resistance of a bridge steel in chloride atmosphere. Corros. Sci..

[23] Liu Y., Pan H., Zhao Y., Zhou L., Feng J., Jiang Y. (2024). New insights for composition design: A novel synergistic corrosion-resistant effect of Fe–Mn–Al–Cr–Si–Mo–C lightweight steel in 3.5 wt% NaCl solution. J. Mater. Res. Technol..

[24] (2021). Metallic Materials—Tensile Testing—Part 1: Method of Test at Room Temperature.

[25] (2020). Metallic Materials—Charpy Pendulum Impact Test Method.

[26] Kozeschnik E., Bhadeshia H.K.D.H. (2008). Influence of silicon on cementite precipitation in steels. Mater. Sci. Technol..

[27] Mingjie S., Baojian H., Tao Y. (2022). Micromechanical simulations and experimental characteristics of randomly distributed carbon nanotubes reinforced Mg matrix composites. J. Alloys Compd..

[28] Sarac B., Sharifikolouei E., Zheng Y.H., Yüce E., Asci A., Keckes J., Sarac A.S., Eckert J. (2025). Electrochemical impedance behavior and corrosion resistance of amorphous 316-type stainless steel microfibers in saline environment. Mater. Today Commun..

[29] Zhao Q.C., Luo H., Li C.T., Yan B.B., Liang G.X. (2026). Examining the passive properties of laser powder bed fused austenitic, stainless steel in hydrochloric acid: Effect of building directions. Constr. Build. Mater..

[30] Wang D.S., Sun X.H., Liu W., Wang B., Sun S.B., Jiang Y.C., Chang X.T. (2025). Effect of ammonium ions on the corrosion behaviors of high-manganese austenitic steel. Int. J. Electrochem. Sci..

[31] Zhou Y.L., Chen J., Liu Z.Y. (2013). Corrosion Behavior of Rusted 550 MPa Grade Offshore Platform Steel. J. Iron Steel Res. Int..

[32] Refait P., Jeannin M., Urios T., Fagot A., Sabot R. (2019). Corrosion of low alloy steel in stagnant artificial or stirred natural seawater: The role of Al and Cr. Mater. Corros..

[33] Zhang D., Zhang X., Wang S., Zhang F., Ren H., Zhang J., Yan Q. (2026). A comparative study on the corrosion resistance and microstructure stability of Fe-Ni-Cr-Al-Si based austenitic steel in lead-bismuth eutectic with 10^−6^ wt.% oxygen at 600 and 650 °C for up to 6000 h. J. Nucl. Mater..

[34] Dai C., Luo H., Li J., Du C.W., Liu Z.Y., Yao J.Z. (2020). X-ray photoelectron spectroscopy and electrochemical investigation of the passive behavior of high-entropy FeCoCrNiMox alloys in sulfuric acid. Appl. Surf. Sci..

[35] Kimura M., Kihira H., Ohta N., Hashimoto M., Senuma T. (2005). Control of Fe(O,OH)_6_ nano-network structures of rust for high atmospheric-corrosion resistance. Corros. Sci..

[36] Yang G., Wang L., Fu X.Q., Ji Y.C., Cao W.Q., Wang C.Y., Liang J.X., Dong C.F. (2025). Enhancing the corrosion resistance of low-density steels via Al-rich oxide film formation by grain size optimization. Corros. Sci..

[37] Fajardo S., Llorente I., Jiménez J.A., Bastidas J.M., Bastidas D.M. (2019). Effect of Mn additions on the corrosion behaviour of TWIP Fe-Mn-Al-Si austenitic steel in chloride solution. Corros. Sci..

[38] Zhang L.B., Ning F.Q., Yan H., Liu J., Wang H. (2025). The effect of Si on the microstructure and corrosion resistance of XSi9Cr ferritic/martensitic steel in oxygen-saturated lead-bismuth eutectic. Corros. Sci..

[39] Eijk C.v.d., Dalaker H., Safarian J. (2023). Possibilities and Limitations of the Use of Hydrogen in Different Metallurgical Sectors. Mater. Proc..

[40] Lins V.F.C., Freitas M.A., Silva E.M.P.E. (2004). Corrosion resistance study of Fe–Mn–Al–C alloys using immersion and potentiostatic tests. Appl. Surf. Sci..

[41] Monnier J., Burger E., Berger P., Neff D., Guillot I., Dillmann P. (2011). Localisation of oxygen reduction sites in the case of iron long term atmospheric corrosion. Corros. Sci..

[42] Chinara M., Ghosh R., Mukherjee S., Mondal K. (2025). Corrosion mechanism of line pipe steels (API X70 and X80 grades) under aggressive salt-spray exposure. Mater. Chem. Phys..

[43] Li Z.L., Song J.L., Chen J.H., Yu Q., Xiao K. (2023). Corrosion behavior of a high-strength steel E690 in aqueous electrolytes with different chloride concentrations. J. Mater. Res. Technol..

[44] Melchers R.E. (2004). Effect of small compositional changes on marine immersion corrosion of low alloy steels. Corros. Sci..

